# The impact of thought probes and other encoding interruptions on memory

**DOI:** 10.1177/17470218251398503

**Published:** 2025-11-07

**Authors:** Dillon H. Murphy, Gene A. Brewer

**Affiliations:** 1 Department of Psychology, University of California, Riverside, CA, USA

**Keywords:** Mind-wandering, reactivity, memory, thought probes, cognitive load

## Abstract

Whenever we work towards completing a task, such as learning some information, we are susceptible to attentional lapses where our thoughts stray from the demands of the current task to something unrelated (i.e., mind-wandering). Although prior work indicates that the presence of mind-wandering probes (used to measure task-unrelated thoughts) in a cognitive task may not impact the measurement of abilities like processing speed, there could be reactive effects involving memory. We examined whether mind-wandering probes can impact memory by having participants study lists of words to remember for later tests; at pseudo-random intervals during encoding, participants either responded to mind-wandering probes, answered math problems, had unfilled interstimulus intervals, or studied the lists without any interruptions. Results revealed that mind-wandering probes (or other interruptions) do not significantly impact overall memory performance (though there may be some impact on items immediately preceding or following a probe) or the temporal dynamics of episodic memory. Thus, the present study suggests that using mind-wandering probes introduces minimal unexpected bias into research designs such that these interruptions do not adversely affect or benefit memory performance, consistent with prior research focused primarily on other cognitive domains.

During memory tasks, researchers sometimes interrupt the learning process to examine the cognitive processes potentially contributing to a learner’s ability to remember information. For example, after studying each item in a list, some work solicits predictions of future memory performance for that item (i.e., judgments of learning or JOLs, see Rhodes, 2016). Researchers also sometimes administer thought probes to measure mind-wandering during encoding (e.g., Feng et al., 2013). These interruptions during learning can help provide insights into the cognitive processes contributing to memory, but they may also influence encoding and retrieval. In the present study, we were interested in whether interruptions such as mind-wandering probes during encoding impact memory, as this would have important implications for the design of mind-wandering studies (or other related work) examining encoding and/or retrieval processes.

Whenever we work towards completing a task, such as learning some information, we are susceptible to attentional lapses where our thoughts stray from the demands of the current task to something unrelated (i.e., mind-wandering; see Christoff et al., 2016; Seli, Carriere, et al., 2018; Seli, Kane, et al., 2018), perhaps due to lapses in attentional control (see McVay & Kane, 2010). Instances of mind-wandering can reduce the number of executive/attentional resources available to a learner (see Smallwood & Schooler, 2006), leaving fewer attentional resources available for encoding (see Smallwood, 2010). As such, mind-wandering during encoding (but not at retrieval) is often associated with impaired memory performance (e.g., Garlitch & Wahlheim, 2020; Whitehead et al., 2021; see Blondé et al., 2022 for a review), but there can be both costs and benefits to mind-wandering (Smallwood & Schooler, 2015). For example, mind-wandering has been linked to enhanced creativity, future planning, and problem solving (Baird et al., 2011, 2012; Mooneyham & Schooler, 2013). At the same time, there are interventions aimed at reducing unproductive mind-wandering to improve cognitive and academic performance (e.g., Mrazek et al., 2013; Peterson & Wissman, 2020).

Mind-wandering can be assessed in many ways (see Kane et al., 2021 for an examination of the various measurements of mind-wandering), but the most common method is soliciting thought probes where participants report their conscious experience during an ongoing task (Weinstein, 2018). These probes typically occur within a subset of primary task trials, and researchers can vary the frequency of probes as well as the interval between probes. More frequent mind-wandering is often associated with poor primary-task performance (e.g., McVay & Kane, 2009; Unsworth & McMillan, 2014), and such findings can have important implications in cognitive psychology research. Quantifying participants’ mind-wandering can reveal much about cognitive processes, but it is important to consider whether thought probes alter primary task performance, as this could introduce a source of unaccounted bias.

Previous work has examined whether mind-wandering probes embedded within a primary task influence cognitive performance. For example, Wiemers and Redick (2019) showed no difference in the Sustained Attention to Response Task when participants responded to mind-wandering probes compared to participants not responding to mind-wandering probes. Additionally, Arnicane et al. (2021) showed no difference in performance on a visual working memory task on trials where participants were probed versus trials where participants were not probed. Moreover, other work has shown that probe frequency does not affect task performance (e.g., He & Li, 2023; Robison et al., 2019; Schubert et al., 2020; Seli et al., 2013). Thus, prior work generally suggests that thought probes do not contaminate primary task measures of interest, but episodic memory processes may be uniquely affected compared with the cognitive processes previously examined.

Although prior work has not found evidence that mind-wandering probes impact certain cognitive abilities (e.g., Arnicane et al., 2021; Wiemers & Redick, 2019), prior work suggests that memorial processes can easily be affected by secondary tasks, even something as simple as arbitrarily clicking on to-be-remembered or to-be-forgotten words (e.g., Murphy, Halamish, et al., 2023). Additionally, while learning information, researchers often solicit metacognitive predictions of performance (see Rhodes, 2016 for a review) and these predictions can alter what or how much is remembered (e.g., Arbuckle & Cuddy, 1969; Double & Birney, 2019; Double et al., 2018; Ingendahl et al., 2025; Janes et al., 2018; Kaya & Mulligan, 2024; King et al., 1980; Murphy & Castel, 2021; Murphy, Hoover, et al., 2023; Soderstrom et al., 2015; Spellman & Bjork, 1992; Witherby & Tauber, 2017; Zheng et al., 2024). Accounts of this metacognitive reactivity in memory have suggested that predictions of learning can strengthen the encoding of certain information (Soderstrom et al., 2015), enhance the encoding of item-specific information (Senkova & Otani, 2021), modify attention (Castel et al., 2012), or change a learner’s goals (Mitchum et al., 2016), and metacognitive reactivity may be attributable to several of these mechanisms (see Janes et al., 2018; Myers et al., 2020). Thus, similar to making metacognitive judgments, the presence of responding to thought probes (or other types of interruptions) could similarly alter how we remember information.

Despite thought probes not involving the learner assessing any item-specific information, like most metamemory judgments that can cause reactivity, pausing the encoding process could alter rehearsal processes that impact memory. Specifically, one potential reason why mind-wandering probes could impact memory performance is the costs associated with task switching (see Wylie & Allport, 2000). Task switching refers to how participants manage to shift their focus between primary tasks (like encoding words) and intermittent demands (such as responding to mind-wandering probes). In the context of a memory task, participants may be focused on rehearsing the to-be-remembered words and engaging in elaborative encoding strategies (e.g., mental imagery, sentence generation, etc.) to remember them, but periodically stopping participants to assess their cognitive state could disrupt these encoding processes and impair subsequent memory performance.

Prior research has demonstrated that even brief task interruptions can impair memory performance, particularly when they disrupt ongoing encoding or rehearsal processes (Altmann et al., 2014). For example, even unfilled pauses in a task have been shown to impair performance compared to conditions with continuous task flow (Hodgetts & Jones, 2006). Thus, interrupting a memory task, regardless of the interruption’s content, may introduce costs relative to an uninterrupted baseline. At the same time, other research suggests that interruptions may sometimes be beneficial, particularly in contexts involving monotony or sustained attention demands. For example, Ariga and Lleras (2011) found that brief breaks can improve performance by helping participants re-engage with the task. These competing possibilities raise important questions about whether thought probes, or similar interruptions during encoding, impair or enhance memory.

When learners study lists of words, they often rehearse each word by repeating it to themselves sub-vocally, and learners often rehearse several items during a single item’s presentation. For example, when a learner studies the fourth item in a list, study time for this item could be shared with the first three items as well (i.e., cumulative rehearsal) such that learners are rehearsing all five items during this item’s presentation (see Fischler et al., 1970; Rundus, 1971; Rundus & Atkinson, 1970). Since other items may be rehearsed during a given item’s presentation, pausing the memory task to respond to a thought probe could disrupt this process. Specifically, if a learner is studying the eighth item in a list, they may be rehearsing items six and seven as well. However, if the learners respond to a thought probe following the presentation of item eight, this switching from encoding processes to responding to a thought probe could potentially (a) disrupt the rehearsal of item eight and those preceding it if participants do not engage in rehearsal while responding to the probe or (b) could allow for more rehearsal for item eight and those preceding it (because the learner does not have to study new information during the probe, they can simultaneously engage in rehearsal for recently presented items while responding to the probe). Thus, it is possible that mind-wandering probes impact memory, though the effect could be positive or negative.

Furthermore, if a learner is studying a list of to-be-remembered information, reminding the learner of their off-task thoughts via mind-wandering probes could change encoding operations and alter memory performance. If mind-wandering probes serve to catch people the moment that they are experiencing an attentional lapse, people could use that information as a cue telling them they need to focus and try harder to be “on task”; hence the mind-wandering probes could serve to reengage participants and subsequently enhance memory (perhaps further justifying instructors giving students frequent breaks during long lectures; see Smallwood et al., 2007 for a discussion of the educational implications of mind-wandering).

If mind-wandering probes improve performance on a memory task, this could reveal a potentially useful technique for educators (see Blondé et al., 2022 for a review of mind-wandering effects on memory as well as a potential benefit of mind-wandering). However, it is also possible that thought probes increase the frequency of mind-wandering by drawing participants’ attention to their off-task thoughts, resulting in poor memory performance (learners would then need to re-engage in the memory task). Alternatively, mind-wandering probes may not impact memory processes, which would be ideal for mind-wandering studies examining memory performance (e.g., Feng et al., 2013). Together, these competing hypotheses suggest that elucidating whether procedures that intrude on the encoding process, such as mind-wandering probes, impact memory performance is imperative for directing future research and interpreting prior work.

## The current study

In the current study, we examined whether including thought probes or other interruptions during encoding produces reactive effects on memory performance. Across two experiments, participants studied lists of words for later free recall while periodically experiencing one of several types of interruptions: mind-wandering probes, math problems, unfilled inter-stimulus intervals (ISIs, Experiment 1), or no interruptions (Experiment 2). By comparing recall performance and retrieval dynamics across these conditions, we aimed to determine whether thought probes impair encoding or alter the temporal structure of memory.

## Experiment 1

In Experiment 1, we examined whether mind-wandering probes or other interruptions impact memory performance during word list learning. Specifically, participants were presented with lists of 50 to-be-remembered words, and each word was studied for 3 s. Following the study phase, there was a 30-s delay where participants were asked to count backwards from a random number by 7 s. After the delay, participants completed a 2 min free recall test. This process was repeated for five lists of words. Crucially, throughout each list, participants encountered five interruptions, occurring at pseudo-random intervals. During these interruptions, some participants responded to mind-wandering probes. As a comparison, other participants answered math problems (reporting the product of 3 digits) while studying each list at an identical frequency and duration as participants responding to mind-wandering probes. Finally, a third group of participants had an ISI in place of probes/math problems (i.e., a “do nothing” condition). We examined overall memory performance, how thought probes/math problems/ISIs impact memory for items immediately preceding and following the interruption, and how participants transitioned between items during recall as a proxy for learners’ temporal-contextual binding during encoding.

### Method

#### Participants

Participants were 308 undergraduate students (*M*_age_ = 18.95, *SD*_age_ = 1.84) recruited from the Arizona State University (ASU) Human Subjects Pool. Participants were tested online and received course credit for their participation. Participants were excluded from analysis if they admitted to cheating (e.g., writing down answers) in a post-task questionnaire (they were told they would still receive credit if they cheated). This exclusion process resulted in zero exclusions. Participants were also excluded if they did not attest to giving their best effort on the task. This exclusion criterion resulted in zero exclusions. We aimed to collect around 100 participants per condition. Given that this work was exploratory, the sample size was selected based on the expectation of detecting a medium effect size. Informed consent was acquired, and the study was completed per the ASU Institutional Review Board (Online Cognitive Ability Testing: STUDY00018136).

#### Materials

The words on each list were randomly selected from a pool of 690 unrelated words (e.g., button, chart, twig) that were between 4 and 7 letters (*M* = 4.85, *SD* = 0.99). On the log-transformed Hyperspace Analogue to Language frequency scale (with lower values indicating lower frequency in the English language and higher values indicating higher frequency), words ranged from 4.73 to 14.35 (*M* = 9.48, *SD* = 1.57). In terms of concreteness (with lower values indicating lower concreteness and higher values indicating higher concreteness), words ranged from 1.19 to 5.00 (*M* = 4.16, *SD* = 0.84). Frequency and concreteness ratings were generated using the English Lexicon Project website (Balota et al., 2007). Words are available on OSF, and the data from several pilot studies are also available on OSF. The task was programmed and administered using Collector, an open-source web-based platform for creating and hosting psychology experiments; the full source code and documentation are available at https://github.com/gikeymarcia/Collector.

#### Procedure

At the beginning of the task, participants were asked to (a) be prepared to complete the study in a single, 30-min session (i.e., no prolonged breaks), (b) complete this study in an environment with minimal distraction (i.e., put away extra devices, close unneeded tabs, and turn off any music or TV in the background), (c) complete the study on a laptop or desktop computer (i.e., do NOT use a mobile phone or a tablet), and (d) if in the presence of other people, try to move somewhere more secluded. Participants were then asked, “Please give your full effort on this task! PLEASE do not use any external aides and thus use ONLY YOUR OWN memory to complete the study.” Then, participants were told, “This task will be very difficult. We do not expect you to remember everything; you will likely only be able to remember some of the information. Please do not cheat by taking pictures of the screen, writing things down, or using any other aid to complete this task.” Finally, we again asked participants that they do not cheat on this task by using any memory aids and they give their full effort on this task. Participants then had to attest that they would not cheat and that they would give their best effort.

Participants were presented with lists of 50 unrelated words to remember for a later test. Words were presented in black, size 48 Times New Roman font on a white background. Participants were told that each list would contain multiple words and that each word would be shown individually for 3 s; however, they were not informed of the exact number of words per list. Words were presented continuously with no ISI; a new word appeared on screen every 3,000 ms (i.e., as soon as the previous word disappeared). After the presentation of all words, participants completed a 30-s distraction task where they were given a random number greater than 500 and asked to count backwards by seven. This task was self-paced and unmonitored—participants were not required to input responses, count aloud, or report the final number reached after the 30-s interval.

Following the distractor task, participants were asked to recall all the words they could remember from the just-studied list. At the start of the recall phase, a text box appeared on screen along with the instruction: *“Please type all of the words that you can remember from the just-presented list in the box below. Please try to continue to recall words throughout the entire test phase.”* The recall period lasted 2 min, after which participants were prompted to click a button (“Next”) to begin the next list; thus, the transition to the next list was participant-initiated, not automatic. This was repeated for a total of five study-test cycles.

Throughout the task, one group of participants (*n* = 104) was periodically asked about their level of attentiveness. Specifically, they were asked to characterize their current conscious experience; participants indicated whether they were either (a) totally focused on the current task (i.e., paying attention to the word pairs and trying to learn them), (b) thinking about their performance on the task or how long it is taking (i.e., whether they will remember the information but also wondering whether the task will end soon), (c) distracted by information present in the room (e.g., sights and sounds), (d) zoning out/mind-wandering (i.e., their attention has shifted away from the task to self-generated thoughts unrelated to the task), or (e) “Other” (taken from Unsworth & McMillan, 2014). During mind-wandering probes, all five response options were displayed on screen simultaneously and in a fixed order for all participants. Each option was preceded by an open circle that participants could click to indicate their response; once selected, the circle filled in. Participants could select only one response option per probe. Responses were recorded automatically after 7 s, regardless of whether a selection was made (participants did not respond 7.5% of the time), and then the task automatically resumed. We embedded five mind-wandering probes on each list, and the probes occurred at pseudo-random intervals.

As a comparison, in place of the mind-wandering probes, another group of participants (*n* = 101) was asked to answer math questions at an identical frequency and duration (the math problem lasted 7 s whether participants answered or not; participants did not respond 30.1% of the time) as participants responding to mind-wandering probes. Participants in the math group were periodically shown a three-digit number (e.g., “942”) and were instructed to type the product of the three digits (e.g., 9 × 4 × 2 = 72) into a text box. The math phase lasted 7 s regardless of whether a response was entered; responses were automatically recorded when the time elapsed. Digits for the math problems were randomly generated. Finally, another group of participants (*n* = 103) was given an ISI at an identical frequency and duration as the probes and math problems.

Participants were not given practice trials in any condition, but they were informed in advance about the nature and timing of the interruptions. In the mind-wandering probe condition, participants were told that they would periodically report their attentional state and were given detailed descriptions of the response options. In the math condition, participants were told they would periodically be asked to solve three-digit multiplication problems, with an example provided. In the ISI condition, participants were told to expect periodic 7-s breaks between words. All participants were informed that each interruption would last 7 s and, when applicable, were instructed to respond quickly.

Interruptions were visually distinct in that a study word did not appear during those trials. In the ISI condition, the screen was left blank for 7 s. In the math condition, a math problem appeared in place of a word (e.g., “942”), and in the mind-wandering condition, the attentional probe and response options were displayed on screen. No special background colors or auditory cues were used; the absence of a to-be-remembered word served as the primary signal that an interruption was occurring. Interruptions were inserted at fixed pseudo-random serial positions that were consistent across participants/conditions. To generate these positions for each list, we used Excel to generate random positions for the interruptions. Two constraints were applied to ensure meaningful before-and-after comparisons: probes could not appear in the first or last serial position and could not occur on back-to-back trials.

### Results

Reliability information (Cronbach’s α) for the primary variables of interest can be found in Table 1. To examine mind-wandering rates, we first coded participants’ responses to the mind-wandering probes as either mind-wandering (distracted by information present in the room (sights and sounds) or zoning out/mind-wandering) or not mind-wandering (totally focused on the current task/thinking about their performance on the task or how long it is taking); “Other” responses were not considered mind-wandering or not mind-wandering (“Other” responses occurred less than 1% of the time). We then computed the proportion of the time participants indicated that they were mind-wandering. On average, participants indicated that they were mind-wandering 35% of the time (*SD* = 27%). In terms of performance on the math problems, participants responded correctly 39% of the time (*SD* = 27%).^1^ For participants responding to mind-wandering probes, mind-wandering rate correlated with recall [*r*(102) = −.35, *p* < .001, 95% CI [−0.51, −0.17] such that the more participants mind-wandered, the less they recalled. Additionally, performance on the math problems was positively related to recall [*r*(99) = .44, *p* < .001, [0.26, 0.58] such that the more math problems a participant answered correctly, the more words they recalled.

**Table 1.** Reliabilities (Cronbach’s α) between lists for the primary variables of interest in Experiments 1 and 2.

Variable	Cronbach’s α
Experiment 1	
Recall	.87
Recall—words before interruption	.58
Recall—words after interruption	.50
Mind-wandering rate	.85
Math accuracy	.89
Experiment 2	
Recall	.88
Recall—words before interruption	.61
Recall—words after interruption	.58
Mind-wandering rate	.90

**Figure 1.**
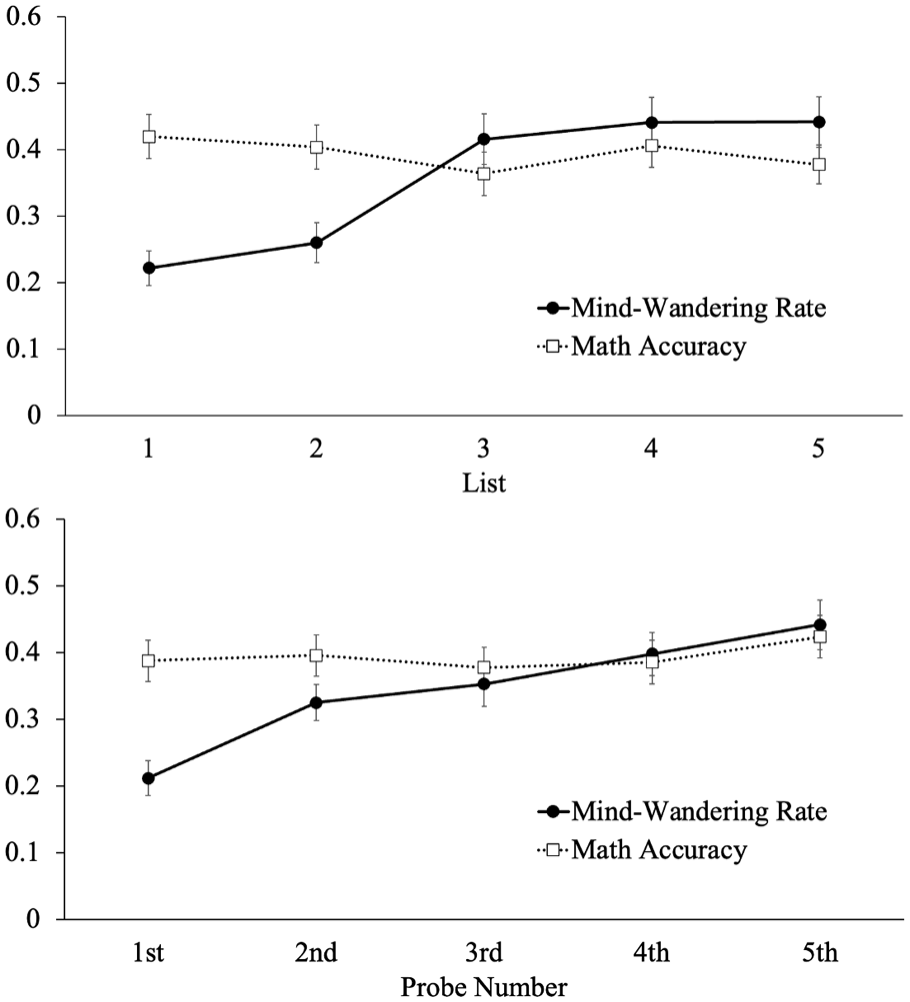
Mind-wandering rate and math accuracy as a function of list (top) and probe number within each list in (bottom) Experiment 1. *Note.* Error bars reflect the standard error of the mean. We note that the pattern of results in Experiment 2 was similar to Experiment 1 (as shown here).

We also analyzed mind-wandering rate and math accuracy as a function of list (see Figure 1, top panel). Specifically, we conducted a logistic multi-level model (items nested within participants) with mind-wandering rate modeled as a function of list. Results revealed that mind-wandering increased as the task endured [e^B^ = 1.50, 95% CI [1.39, 1.62], *z* = 10.47, *p* < .001]. However, a similar model on math accuracy revealed that performance on the math problems did not differ throughout the task [e^B^ = 0.95, [0.89, 1.02], *z* = −1.41, *p* = .159]. Thus, mind-wandering increased as the task endured, but performance on the math problems was relatively stable.

Similarly, we examined mind-wandering rate and math accuracy as a function of ordinal position within each list (i.e., the first probe vs. the second probe vs. the third probe, etc.; see Figure 1, bottom panel). Specifically, we conducted a logistic multi-level model (items nested within participants) with mind-wandering rate modeled as a function of probe number within each list. Results revealed that mind-wandering increased as each list endured [e^B^ = 1.39, 95% CI [1.29, 1.50], *z* = 8.77, *p* < .001]. However, a similar model on math accuracy revealed that performance on the math problems did not differ as a function of position within the list [e^B^ = 1.04, [0.97, 1.11], *z* = 1.07, *p* = .287]. Thus, participants were less likely to mind-wander at the beginning of a list, but mind-wandering increased throughout the presentation of each list while math accuracy was consistent throughout each list.

**Figure 2.**
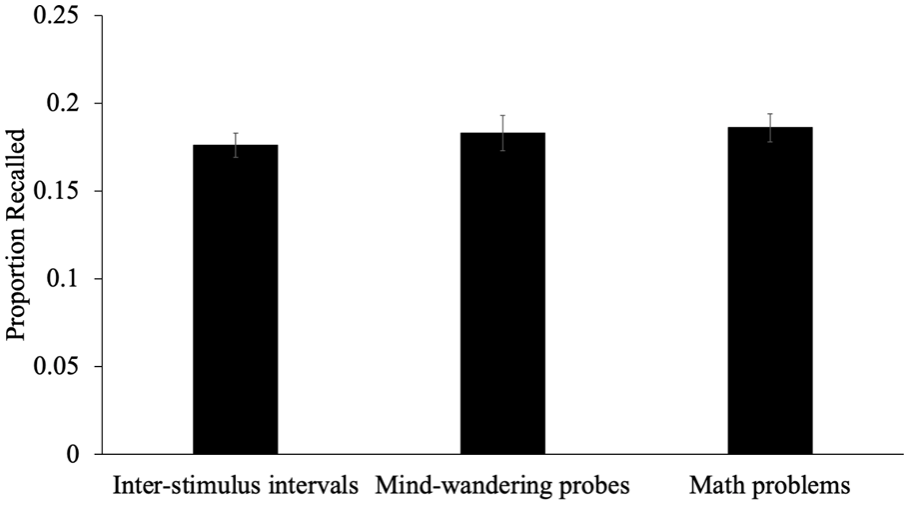
The proportion of words recalled as a function of whether participants responded to mind-wandering probes, answered math problems, or had an ISI during the study phase in Experiment 1. *Note.* Error bars reflect the standard error of the mean.

To examine differences in recall between groups (see Figure 2), we conducted a one-way ANOVA. Results revealed that the proportion of words recalled for participants responding to mind-wandering probes (*M* = 0.18, *SD* = 0.10), participants answering math problems (*M* = 0.19, *SD* = 0.08), and participants with ISIs (*M* = 0.18, *SD* = 0.07) was similar [*F*(2, 305) = 0.35, *p* = .706, η_p_^2^ < .01]. To further investigate this potential effect, we computed Bayes Factor compared to a null model using JASP (Love et al., 2019; for information on interpreting Bayes factors, see Kass & Raftery, 1995). A Bayesian ANOVA using the default priors on JASP provided strong evidence that there were no differences in recall between the three groups [BF_01_ = 20.73]. To provide further evidence, we also calculated credible intervals (CrI_95%_) for the effect of each group in the Bayesian model. In Bayesian statistics, a credible interval is a range of values in which an unknown parameter falls with a certain probability, reflecting the degree of certainty or “credibility” we have about the parameter’s true value. The credible interval for participants responding to mind-wandering probes [CrI_95%_ = −0.01–0.01], participants answering math problems [CrI_95%_ = −0.01–0.02], and participants with ISIs [CrI_95%_ = −0.02–0.01] all included 0, further suggesting that recall was similar in each of these groups.

**Figure 3.**
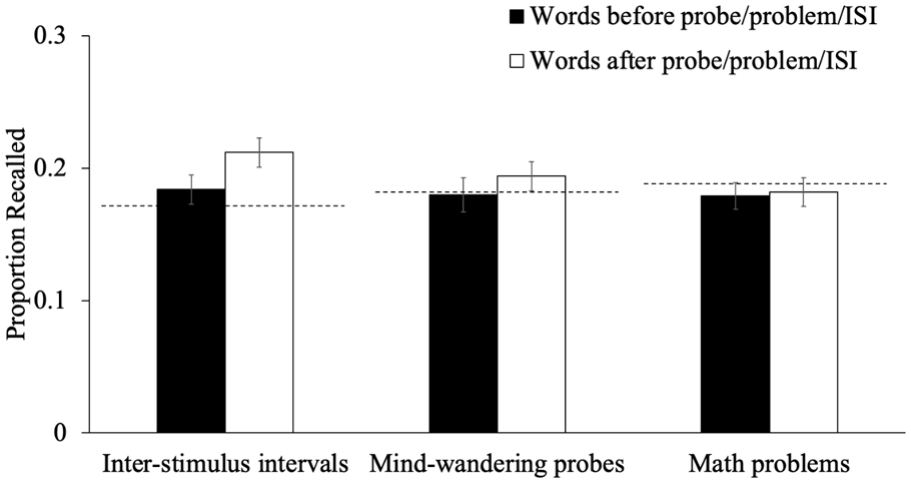
The proportion of words recalled that were studied immediately before or after the mind-wandering probe/math problem/ISI as a function of whether participants responded to mind-wandering probes, answered math problems, or had an ISI during the study phase in Experiment 1. *Note.* Dashed lines represent the average recall for words not immediately before or after interruptions for each group. Error bars reflect the standard error of the mean. ISI = inter-stimulus interval.

Next, we examined recall for words studied immediately before and immediately after the mind-wandering probe, math problem, or ISI (see Figure 3). A 3 (timing: item immediately before interruption, item immediately after interruption, baseline item) × 3 (group: mind-wandering probe, math problem, ISI) mixed-factor ANOVA to examine whether the recall of items surrounding interruptions differed across groups. There were not group differences in recall [*F*(2, 305) = 0.13, *p* = .881, η_p_^2^ < .01, BF_01_ = 16.35]. However, there was an effect of timing [*F*(2, 610) = 5.57, *p* = .004, η_p_^2^ = .02, BF_10_ = 2.57], and this effect was qualified by a significant interaction between timing and group [*F*(4, 610) = 3.28, *p* = .011, η_p_^2^ = .02, BF_10_ = 1.33] such that, in the ISI condition, recall for items presented after the interruption was higher than baseline recall [*p*_holm_ < .001, *d* = 0.40]—all other comparisons were not significant [all *p*s > .441]. This suggests that memory benefits can emerge specifically in the ISI condition, where the interruption is undemanding and does not require a task switch. Such a benefit may reflect the formation of a new temporal context or event boundary, allowing the first item after the ISI to receive enhanced encoding as a kind of local primacy item. This is consistent with the idea that unfilled intervals may afford participants time to reset attention, whereas interruptions that require engagement with a secondary task (e.g., math or probes) may not provide the same opportunity for post-interruption rehearsal or context reinstatement.

**Figure 4.**
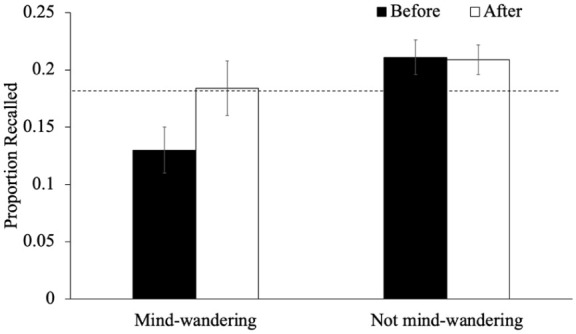
The proportion of words recalled that were studied immediately before or after a given mind-wandering probe as a function of whether participants reported mind-wandering in Experiment 1. *Note.* The dashed line represents the average recall for words not immediately before or after an interruption. Error bars reflect the standard error of the mean.

As an exploratory analysis, we also examined recall performance for words immediately preceding or following an interruption as a function of participants’ responses during said interruption. In terms of mind-wandering, we examined recall for words studied immediately before and immediately after a given mind-wandering probe as a function of whether participants reported mind-wandering (see Figure 4). A 2 (timing: items right before the probe/items right after the probe) × 2 (mind-wandering, not mind-wandering) within-subjects ANOVA revealed an effect of timing such that the recall of items after a mind-wandering probe was better than the recall of items before a mind-wandering probe [*F*(1, 87) = 4.72, *p* = .033, η_p_^2^ = .05, BF_10_ = 0.73]. Additionally, recall was worse when participants had reported mind-wandering [*F*(1, 87) = 7.20, *p* = .009, η_p_^2^ = .08, BF_10_ = 4.13]. The interaction between timing and mind-wandering did not reach significance [*F*(1, 87) = 3.50, *p* = .065, η_p_^2^ = .04, BF_01_ = 0.82] but an exploratory analysis of the simple effects demonstrated that when participants were not mind-wandering, the recall of items preceding and following a probe was similar [*p*_holm_ = .991, *d* = 0.01] but when participants reported mind-wandering, the item directly following the probe was better recalled than the item preceding the probe [*p*_holm_ = .046, *d* = 0.30]. Particularly, a one-sample *t*-test indicates that, when mind-wandering, recall for words before the probe was impaired relative to their baseline performance (0.18), [*t*(87) = −2.50, *p* = .014, *d* = −0.27, BF_10_ = 2.22] but recall after the probe returned to baseline [*t*(87) = 0.18, *p* = .859, *d* = 0.02, BF_01_ = 8.36]. This indicates that the probe may have served as a reminder for participants who were mind-wandering to get back on task.

**Figure 5.**
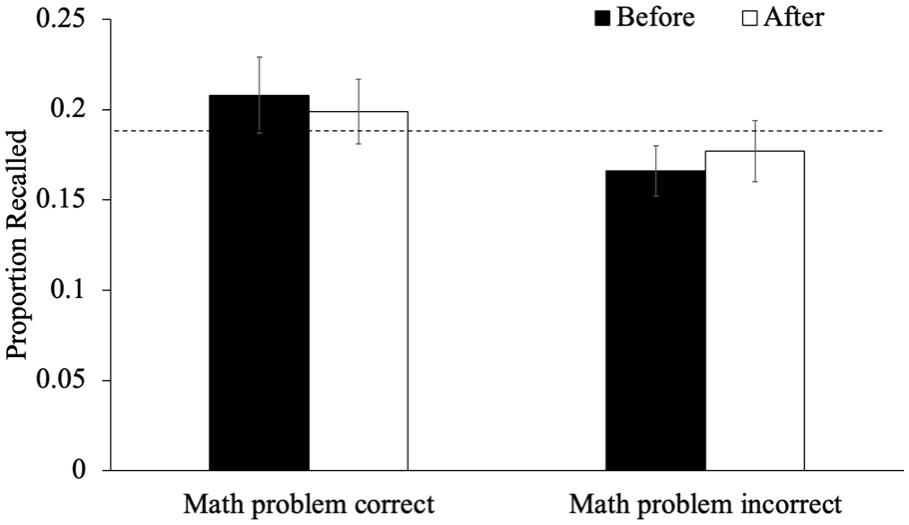
The proportion of words recalled that were studied immediately before or after a given math problem as a function of whether participants answered the problem correctly in Experiment 1. *Note.* The dashed line represents the average recall for words not immediately before or after an interruption. Error bars reflect the standard error of the mean.

In terms of the math problems, we examined recall for words studied immediately before and immediately after a given math problem probe as a function of whether participants answered correctly (see Figure 5). A 2 (timing: items right before the math problem, items right after the math problem) × 2 (correct, incorrect) within-subjects ANOVA did not reveal an effect of timing [*F*(1, 82) = 0.01, *p* = .933, η_p_^2^ < .01, BF_01_ = 7.09]. Additionally, there was no effect of correctness [*F*(1, 82) = 2.91, *p* = .092, η_p_^2^ = .03, BF_01_ = 1.91]. The interaction between timing and math problem accuracy was not significant [*F*(1, 82) = 0.44, *p* = .510, η_p_^2^ = .01, BF_01_ = 4.66].

Finally, we were interested in whether the interruptions (mind-wandering probes, math problems) influence the dynamics of episodic memory retrieval (see Murphy & Brewer, 2025). Specifically, interruptions during encoding may disrupt the lag-recency effect—the use of recently recalled items to aid the retrieval of words presented close together in the encoding phase via the utilization of shared contextual features (Sederberg et al., 2010; Spillers & Unsworth, 2011). This pattern is captured by lag conditional-response probabilities (lag-CRPs; Kahana, 1996). Previous work suggests that learners are more likely to recall adjacent items compared to more distant items and that CRPs are larger in the forward direction compared with the backward direction (Kahana, 1996).

**Figure 6.**
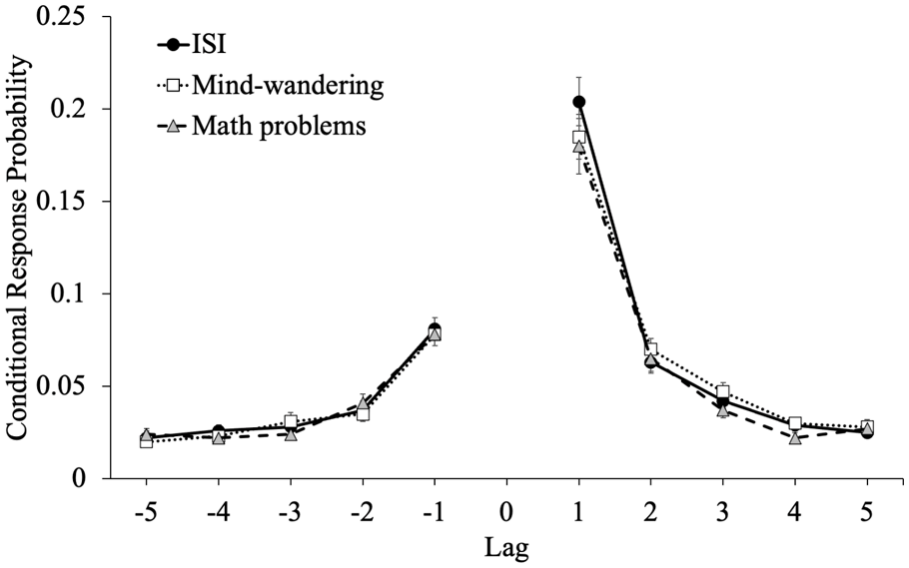
Conditional-response probability functions for each group as a function of lag in Experiment 1. *Note.* Error bars reflect the standard error of the mean.

We calculated lag-CRPs to examine whether disruptions during encoding impact participants’ use of a word’s accompanying temporal and contextual information from the study phase to retrieve additional items. In this measure of how participants transition between responses during retrieval, lag is the ordinal distance between successively recalled items (i.e., the lag between items 4 and 8 would be 4), and the sign of the lag indicates the direction of recall: positive values indicate a forward transition and negative values indicate a backward transition. The CRP for a recall transition illustrates the likelihood that a word from serial position *i* + lag is recalled directly after a word from serial position *i*. The probability of recalling an item from serial position x followed by the item from position x + lag is shown in Figure 6.

To examine differences in the lag-recency effect as a function of attention at encoding, we conducted a 5 (lag: *1–5*; within-subjects factor) × 2 (direction: *forward vs. backward*) × 3 (group: mind-wandering probes, math problems, ISIs) mixed-factor ANOVA. Results revealed that participants showed a forward preference for the direction of transitions [*F*(1, 304) = 173.74, *p* < .001, η_p_^2^ = .36] but this did not differ between groups [*F*(2, 304) = 0.55, *p* = .576, η_p_^2^ < .01]. Additionally, participants showed strong adjacency effects [Mauchly’s *W* = 0.20, *p* < .001; Huynh-Feldt corrected results: *F*(2.10, 639.15) = 355.32, *p* < .001, η_p_^2^ = .54] but lag also did not interact with group [*F*(4.21, 639.15) = 0.90, *p* = .466, η_p_^2^ = .01]. There was an interaction between direction and lag [Mauchly’s *W* = 0.24, *p* < .001; Huynh-Feldt corrected results: *F*(2.18, 662.24) = 85.80, *p* < .001, η_p_^2^ = .22] such that transitions of lag 1 were more likely in the forward direction but there was not a three-way interaction between direction, lag, and group [*F*(4.36, 662.24) = 0.41, *p* = .818, η_p_^2^ < .01]. Moreover, there was no main effect of group [*F*(2, 304) = 0.96, *p* = .383, η_p_^2^ = .01] such that the lag-recency effect was similar whether responding to mind-wandering probes, answering math problems, or experiencing an ISI.

### Discussion

The results of Experiment 1 suggest that interruptions during encoding—whether in the form of mind-wandering probes, math problems, or passive ISIs—do not significantly influence overall memory performance. Specifically, recall was comparable across conditions, and no group differences emerged in the temporal dynamics of retrieval. However, when participants reported mind-wandering, recall for items presented immediately *before* the probe was impaired, suggesting that attentional lapses likely occurred during the encoding of those items, thereby impairing their ability to recall them later. Following the probe, participants’ recall returned to baseline, indicating that the probe may have helped participants refocus attention. These findings suggest that mind-wandering probes can be used to measure attentional state during encoding with minimal concern for reactivity, but the timing and content of attentional lapses—not simply the presence of probes—may selectively influence memory.

## Experiment 2

To address the primary limitation of Experiment 1—the lack of a true no-interruption control—we conducted a follow-up study that retained the mind-wandering probe condition but replaced the math problem and ISI conditions with a no-interruption condition where participants studied the word lists without interruptions during encoding (i.e., the mind-wandering probes were simply skipped); however, the lack of interruptions meant that the study phase was 35 s (5 probes that lasted 7 s each) shorter than the mind-wandering condition. Thus, to control for total task duration, we also included a second, timing-matched no-interruption condition in which the study phase was uninterrupted, but an additional 35-s distractor task was added at the end of the study phase to match the length of the encoding phase for participants in this condition and the probe condition. These additions allowed us to directly assess whether the presence of interruptions, particularly thought probes, impacts memory, relative to a continuous, uninterrupted encoding experience. We hypothesized that memory performance would not significantly differ when responding to mind-wandering probes and having no interruptions.

### Method

#### Participants

Participants were 313 undergraduate students (*M*_age_ = 19.43, *SD*_age_ = 2.39) recruited from the University of California, Riverside (UCR) Human Subjects Pool. Two participants were excluded for cheating, and one was excluded for not attesting that they would give their full effort. Informed consent was acquired, and the study was completed per the UCR Institutional Review Board (Online Cognitive Ability Testing: 30556).

#### Materials and procedure

The procedure for Experiment 2 was similar to that of Experiment 1. As in Experiment 1, participants completed five study-test cycles, each consisting of a list of 50 unrelated words (presented for 3 s each), followed by a 30-s backward-counting distractor task, and then a 2-min free recall test. In the mind-wandering probe condition (identical to Experiment 1; *n* = 106), participants encountered five pseudo-randomly timed thought probes per list, each lasting 7 s and asking participants to report their attentional state. In the standard no-interruption condition (*n* = 106), participants studied each list continuously without any interruptions. In the timing-matched no-interruption condition (*n* = 101), participants also experienced uninterrupted encoding, but the post-list distractor task was extended to 65 s (30 s + 35 s) to match the total duration of the probe condition. All other instructions, materials, and response formats were consistent with those used in Experiment 1.

### Results

Reliability information (Cronbach’s α) for the primary variables of interest can be found in Table 1. On average, participants indicated that they were mind-wandering 33% of the time (*SD* = 28%). For participants responding to mind-wandering probes, mind-wandering rate correlated with recall [*r*(102) = −.33, *p* < .001, 95% CI [−0.49, −0.15] such that the more participants mind-wandered, the less they recalled. As in Experiment 1, we analyzed mind-wandering rate as a function of list and ordinal position within each list. Mind-wandering increased as the task endured [e^B^ = 1.35, [1.25, 1.45], *z* = 7.84, *p* < .001] and as each list endured [e^B^ = 1.47, [1.36, 1.58], *z* = 9.82, *p* < .001]. Thus, participants mind-wandered more on later lists and were more likely to mind-wander towards the end of each list.

**Figure 7.**
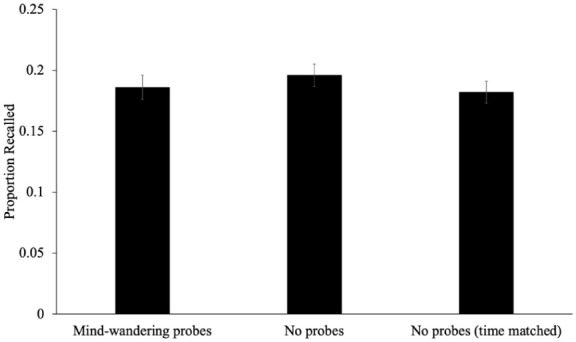
The proportion of words recalled as a function of whether participants responded to mind-wandering probes, had no mind-wandering probes, or had no mind-wandering probes but an extended delay to match encoding time in Experiment 2. *Note.* Error bars reflect the standard error of the mean.

To examine differences in recall between groups (see Figure 7), we conducted a one-way ANOVA. Results revealed that the proportion of words recalled for participants responding to mind-wandering probes (*M* = 0.19, *SD* = 0.11), participants with no interruptions (*M* = 0.20, *SD* = 0.09), and participants with no interruptions but encoding time matched (*M* = 0.18, *SD* = 0.10) was similar [*F*(2, 310) = 0.52, *p* = .593, η_p_^2^ < .01, BF_01_ = 17.91]. The credible interval for participants responding to mind-wandering probes [CrI_95%_ = −0.02–0.01], participants with no interruptions [CrI_95%_ = −0.01–0.02], and participants with no interruptions but encoding time matched [CrI_95%_ = −0.02–0.01] all included 0, further suggesting that recall was similar in each of these groups.

**Figure 8.**
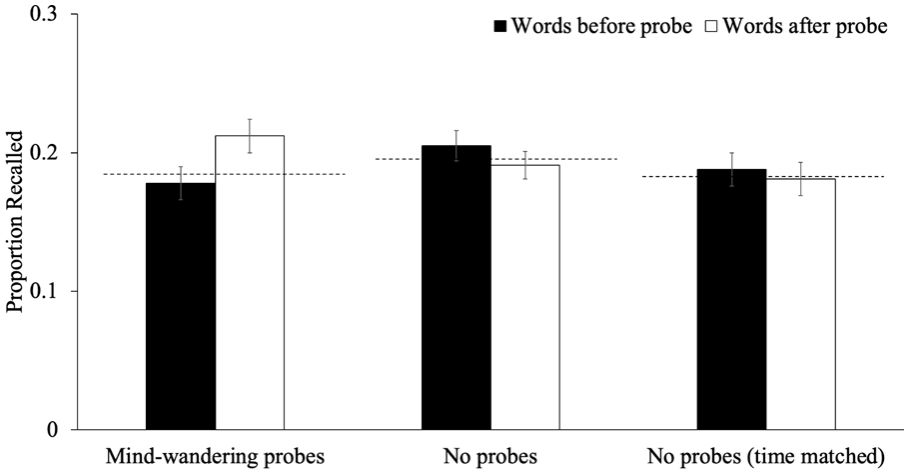
The proportion of words recalled that were studied immediately before or after the mind-wandering probes, as well as recall for the words in the same positions in the other conditions in Experiment 2. *Note.* The dashed line represents the average recall for words not immediately before or after a probe (or where the probe would have been). Error bars reflect the standard error of the mean.

Next, we examined recall for words studied immediately before and immediately after the mind-wandering probe, or where the probe would have been in the other conditions (see Figure 8). A 3 (timing: item immediately before interruption, item immediately after interruption, baseline item) × 3 (group: mind-wandering probes, no-interruption, timing-matched no-interruption) mixed-factor ANOVA to examine whether the recall of items surrounding interruptions differed across groups. There were not group differences in recall [*F*(2, 310) = 0.45, *p* = .638, η_p_^2^ < .01, BF_01_ = 6.95]. There was an effect of timing [*F*(2, 620) = 1.39, *p* = .250, η_p_^2^ < .01, BF_01_ = 21.50], but there was a significant interaction between timing and group [*F*(4, 620) = 5.82, *p* < .001, η_p_^2^ = .04, BF_10_ = 78.50] such that, in the mind-wandering probe condition, recall for items presented after the interruption was higher than baseline recall [*p*_holm_ = .005, *d* = 0.25] and items presented before the interruption [*p*_holm_ = .006, *d* = 0.31]—all other comparisons were not significant [all *p*s > .999]. Thus, there may be a *facilitative boost* in recall for post-interruption items—possibly due to increased salience or attentional resetting—rather than a disruption of memory for pre-interruption items.

**Figure 9.**
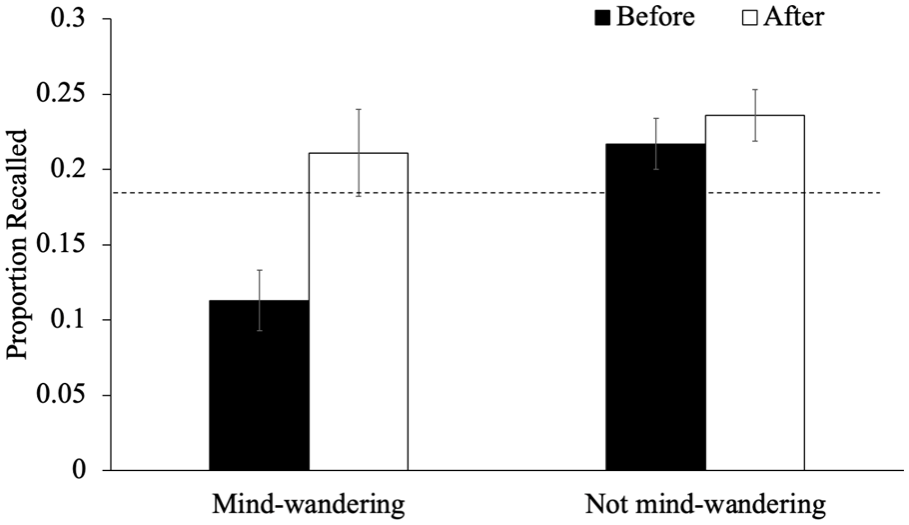
The proportion of words recalled that were studied immediately before or after a given mind-wandering probe as a function of whether participants reported mind-wandering in Experiment 2. *Note.* The dashed line represents the average recall for words not immediately before or after an interruption. Error bars reflect the standard error of the mean.

We next examined recall performance for words immediately preceding or following an interruption as a function of whether participants reported mind-wandering (see Figure 9). A 2 (timing: items right before the probe/items right after the probe) × 2 (mind-wandering, not mind-wandering) within-subjects ANOVA revealed an effect of timing such that words studied right after a mind-wandering probe were better recalled than words studied right before a mind-wandering probe [*F*(1, 92) = 10.78, *p* = .001, η_p_^2^ = .03, BF_10_ = 7.97]. Additionally, recall was worse when participants had reported mind-wandering [*F*(1, 92) = 9.14, *p* = .003, η_p_^2^ = .04, BF_10_ = 7.46]. The interaction between timing and mind-wandering was significant [*F*(1, 92) = 4.28, *p* = .041, η_p_^2^ = .01, BF_10_ = 2.20]; an analysis of the simple effects demonstrated that when participants were not mind-wandering, the recall of items preceding and following a probe was similar [*p*_holm_ = .882, *d* = 0.10] but when participants reported mind-wandering, the item directly preceding the probe was recalled worse than the item following the probe [*p*_holm_ = .011, *d* = 0.47]. Furthermore, a one-sample *t*-test indicates that, when mind-wandering, recall for words before the probe was impaired relative to their baseline performance (0.18), [*t*(87) = −3.45, *p* < .001, *d* = −0.36, BF_10_ = 26.25] but recall after the probe returned to baseline [*t*(87) = 1.09, *p* = .278, *d* = 0.11, BF_01_ = 4.91]. This suggests that the probes may have helped redirect participants’ attention, enhancing the encoding of subsequent items following a moment of disengagement.

**Figure 10.**
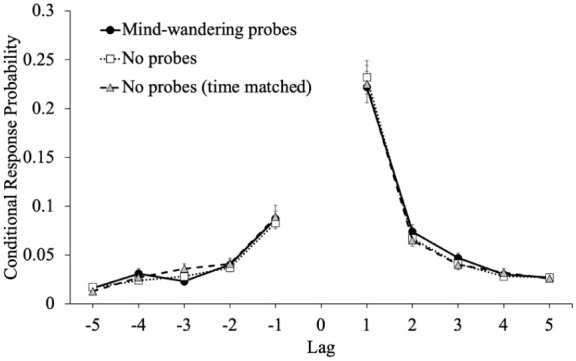
Conditional-response probability functions for each group as a function of lag in Experiment 2. *Note.* Error bars reflect the standard error of the mean.

To examine differences in the lag-recency effect as a function of attention at encoding (see Figure 10), we conducted a 5 (lag: *1–5*; within-subjects factor) × 2 (direction: *forward vs. backward*) × 3 (Group: mind-wandering probes, no probes, no probes (time matched)) mixed-factor ANOVA. Results revealed that participants showed a forward preference for the direction of transitions [*F*(1, 310) = 200.44, *p* < .001, η_p_^2^ = .39] but this did not differ between groups [*F*(2, 310) = 0.32, *p* = .726, η_p_^2^ < .01]. Additionally, participants showed strong adjacency effects [Mauchly’s *W* = 0.08, *p* < .001; Huynh-Feldt corrected results: *F*(1.67, 517.98) = 353.08, *p* < .001, η_p_^2^ = .53] but lag also did not interact with group [*F*(3.34, 517.98) = 0.18, *p* = .925, η_p_^2^ < .01]. There was an interaction between direction and lag [Mauchly’s *W* = 0.09, *p* < .001; Huynh-Feldt corrected results: *F*(1.72, 533.05) = 96.23, *p* < .001, η_p_^2^ = .24] such that transitions of lag 1 were more likely in the forward direction but there was not a three-way interaction between direction, lag, and group [*F*(3.44, 533.05) = 0.39, *p* = .788, η_p_^2^ < .01]. Moreover, there was no main effect of group [*F*(2, 310) = 0.16, *p* = .854, η_p_^2^ < .01] such that the lag-recency effect was similar whether there were mind-wandering probes, no mind-wandering probes, or no mind-wandering probes with encoding time equated.

### Discussion

The results of Experiment 2 replicate and extend the findings from Experiment 1 by demonstrating that mind-wandering probes do not impair overall memory performance, even when compared to conditions with uninterrupted encoding. Specifically, recall was comparable across all conditions, including a true no-interruption control and a timing-matched control that equated total task duration. Moreover, participants exhibited similar lag-recency effects across groups, indicating that the temporal dynamics of memory retrieval were not disrupted by the presence of probes. Although items presented immediately after a probe were recalled better than those presented before, this benefit was again strongest when participants had reported mind-wandering, suggesting that the probes may serve to reorient attention, but not enough to boost overall performance. Collectively, these findings support the use of mind-wandering probes during encoding as a minimally disruptive tool for measuring attentional state.

### Combined analysis

To provide a more comprehensive view of how different types of interruptions affect memory, we conducted a combined analysis including all experimental conditions from both Experiment 1 and Experiment 2. Although originally designed as separate experiments, all conditions were independent and methodologically consistent, allowing for meaningful cross-condition comparisons. This approach enables a more robust assessment of how various interruption types—mind-wandering probes, math problems, unfilled pauses, and uninterrupted baselines—influence free recall. A one-way ANOVA was conducted to examine the effect of condition on the proportion of words recalled across all five conditions pooled from Experiments 1 and 2 (note that the mind-wandering probe conditions were identical). There were no differences in total recall between groups [*F*(4, 616) = 0.63, *p* = .642, η_p_^2^ < .01, BF_01_ = 129.28], indicating that the type of interruption—whether a mind-wandering probe, math problem, unfilled pause, or no interruption—did not significantly impact overall memory performance.

## General discussion

In the present study, we had two primary aims: First, to assess whether inserting interruptions—particularly mind-wandering probes—affects memory performance at a global level; and second, to explore theoretical accounts of how interruptions might impair or facilitate encoding processes. Across two experiments, we found that inserting interruptions during encoding—whether in the form of mind-wandering probes, math problems, or unfilled delays—did not significantly impair or improve overall free recall performance (consistent with Murphy & Brewer, 2025). This null effect held across multiple operationalizations, including comparisons between interruption types and against uninterrupted control conditions. However, exploratory analyses revealed modest, localized effects: items presented immediately before or after an interruption were sometimes recalled at slightly different rates, suggesting that momentary attentional shifts may influence nearby encoding. These findings provide evidence that mind-wandering probes, despite their potential to momentarily disrupt ongoing processing, do not meaningfully alter overall memory performance and are thus unlikely to introduce systematic bias in studies that use such probes.

In our first experiment, we presented participants with lists of words to remember for a later test and either had participants respond to mind-wandering probes, answer math problems, or experience an unfilled ISI at pseudo-random intervals during the study phase. Participants completing the math problems or experiencing ISIs served as comparison conditions—the math questions involved responding to a prompt (computing the product of three digits) and should require enough cognitive resources to disrupt rehearsal (similar to responding to a mind-wandering probe). The ISI condition was included as a baseline where participants had no secondary task (responding to probes or math problems) that controls for the total length of the study phase. Additionally, Experiment 2 included a true no-interruption control condition and a timing-matched version to more directly test whether mind-wandering probes impair memory relative to uninterrupted encoding.

Some evidence suggests that the effect of mind-wandering on memory depends on encoding operations (Thomson et al., 2014), and because lapses of attention become more frequent with increased time on task (see Seli et al., 2013), we hypothesized that mind-wandering probes could alter how information is encoded. For example, if participants begin to mind-wander while learning a long list of words, they likely begin engaging in rote rehearsal or simply reading the words as they appear on-screen. However, when participants are probed for mind-wandering, if they had begun engaging in rote rehearsal or just reading the words as their mind began to wander, the mind-wandering probe may get them back to engaging in more elaborative encoding strategies (e.g., mental imagery) that promote memory rather than just continuing with simply reading the words as they appear. Additionally, thought probes could be beneficial since responding to thought probes may increase attentional effort, and increased attentional effort should result in fewer lapses of attention and better performance (see Unsworth et al., 2022).

The present study provided strong evidence that interruptions during encoding—whether in the form of mind-wandering probes, math problems, or unfilled ISIs—did not impair overall memory performance. At the item level, recall for words presented immediately before an interruption was comparable to baseline, whereas recall for words presented immediately after an interruption was slightly higher, particularly in the ISI condition (Figure 3). This pattern suggests that undemanding interruptions (e.g., ISIs) may facilitate memory for subsequently presented material, potentially by supporting consolidation, resetting attention, or introducing a new boundary. Although a small before-and-after difference also emerged for mind-wandering probes (Figure 8), this benefit was not observed for math problems, indicating that the cognitive demands of the interruption play a key role. However, these differences appeared to be small and not big enough to impact overall performance. Furthermore, analyses of the lag-recency effect revealed no differences across groups, suggesting that interruptions did not alter the temporal organization of episodic recall. Collectively, these findings indicate that embedding thought probes or similar interruptions during encoding does not substantially disrupt memory, mirroring prior results in other cognitive domains (e.g., Arnicane et al., 2021; Wiemers & Redick, 2019).

The lack of reactivity observed in the present study could be due to either (a) there truly is no effect of interruptions or (b) mind-wandering probes, while disruptive, also remind the learner to get back on task, resulting in a net-neutral effect on performance. The present data offer support for both interpretations (though we acknowledge they may appear mutually exclusive). Regarding the first interpretation, when participants respond to mind-wandering probes, they engage in task-switching—the cognitive process of shifting attention between competing goals. Task switching is typically associated with costs (e.g., slower response times or increased errors) due to the need to deactivate one task set and activate another (Wylie & Allport, 2000). These effects are consistent with a broader literature on interruptions, which shows that even short, seemingly innocuous disruptions can impair performance by interfering with goal maintenance or working memory (Altmann et al., 2014; Hodgetts & Jones, 2006). However, in the context of the current study, task-switching costs appear to be minimal.

Regarding the interpretation that mind-wandering probes may offset their potential costs by serving as reminders to re-engage with the task (see Unsworth et al., 2024), our results provide partial support. Specifically, if mind-wandering probes had disrupted encoding and impaired memory, but also acted as a reminder to return to task-relevant processing (which we have some evidence for, see Figures 4 and 9), then math problems, which also interrupt encoding but do not carry such a reminder, should have been purely disruptive. In that case, overall recall performance should have been worse in the math condition than in the mind-wandering probe condition. Yet, this was not observed (see Figures 2 and 7), which supports the interpretation that mind-wandering probes are not uniquely disruptive to episodic memory encoding.

When participants reported mind-wandering, recall for the item immediately preceding the probe was impaired, but when they were not mind-wandering, recall for items before and after the probe was similar. This pattern suggests that attentional lapses can impair memory for recently studied items, but that the act of responding to a probe may help redirect attention back to the task. Given that mind-wandering tends to increase over time during sustained tasks (e.g., Seli et al., 2013), probes presented later in the list may be particularly likely to coincide with attentional lapses. In this way, the presence of a probe might help re-establish task focus, consistent with the “Readiness-to-Remember” framework, which posits that successful episodic retrieval depends on preparatory attentional states and goal-directed engagement (Madore & Wagner, 2022). This is also consistent with the findings of Shi et al. (2023), who found that making metacognitive judgments during encoding reduced mind-wandering (the judgments refocused attention on the task), which, in turn, mediated the reactivity effect on memory. Thus, embedded thought probes may have some small memory benefits, but these effects are not big enough to impact overall performance.

Another theoretical benefit of mind-wandering probes (and possibly math problems as well) is that these interruptions may act as temporal contextual markers that enhance memory for items just prior by tagging them with these context retrieval cues that items in the rest of the list would not have (e.g., Kahana, 1996; Swallow et al., 2009). Thus, if the mind-wandering probes or math problems acted as temporal contextual markers, items immediately following these interruptions should have received a memory benefit, but this was not the case. Rather, items before the boundary were impaired, and performance rebounded after the interruption. As such, mind-wandering probes do not seem to serve as beneficial temporal contextual markers but can remind learners to remain on task.

If thought probes were to help performance (which was not the case in the present study), conditions with more probes should result in faster response times, lower variability in response times, and/or higher recall accuracy. However, Greve and Was (2022) suggest that the attentional demands associated with the criterion task may moderate this effect. Specifically, they demonstrated that during a video lecture, participants who only received a single probe were more likely to report a lapse of attention at the end of the lecture (relative to those who received six probes during the lecture). Thus, there may be conditions where thought probes *do* impact memory performance; future work could examine differing mind-wandering probe frequencies, short versus long probe intervals, probing participants at consistent versus random intervals, short versus long lists of to-be-remembered information, the type of probe, the type of memory test, list length, and the nature of the study materials (e.g., a categorized list, see Senkova & Otani, 2021; Soderstrom et al., 2015).

One limitation of the present study is the low overall recall performance (around 20%). This potential floor effect could mask group differences that might otherwise emerge. It could be interesting to investigate whether these results would remain consistent with easier study materials or in conditions where memory performance is enhanced. Future research could also examine how encoding strategies interact with the effects of interruptions on memory. In the present study, we speculated that participants may have spontaneously shifted between strategies such as rote rehearsal and elaborative imagery, and that thought probes might prompt a return to more effortful encoding strategies. While overall recall did not differ across conditions, we observed small, localized effects for items immediately preceding or following interruptions, suggesting that the impact of interruptions may depend on the encoding processes engaged at that moment. Instructing participants to use elaborative strategies like mental imagery might reduce sensitivity to interruptions and help sustain stable recall across list positions. In contrast, reliance on rote rehearsal—potentially more susceptible to disruption from attentional shifts—may amplify differences in recall surrounding an interruption, either through impaired encoding or enhanced salience of subsequent items. Future work could experimentally manipulate encoding strategy to clarify how such factors moderate the influence of interruptions on memory performance. Future work may also benefit by using overt rehearsal procedures whereby participants rehearse words out loud to determine how items are differentially rehearsed as a function of their distance from an interruption (see Tan & Ward, 2000; Ward et al., 2003).

Although our findings suggest that interruptions during encoding do not impair overall memory performance, it is important to consider potential item-level biases introduced by such interruptions. While participants recalled roughly the same number of words across conditions, this global measure may obscure subtle effects on which items are recalled. Specifically, our data revealed that in some conditions (e.g., ISIs), items immediately following an interruption were recalled at higher rates than baseline items, suggesting that interruptions may bias the identity of recalled items without altering total recall capacity. This echoes findings from the broader interruptions literature, where overall task performance may remain stable, but fine-grained metrics—such as resumption lag or sequence disruptions—reveal meaningful differences. In our case, with 50 words per list and limited recall bandwidth, any increased likelihood of recalling post-interruption items may have come at the expense of other, potentially equally memorable, baseline items. Thus, even in the absence of overall performance differences, introducing interruptions may subtly shift which items are rehearsed or retrieved, raising important questions for future research on how reactive effects of encoding interruptions might influence the content—not just the quantity—of episodic memory.

The challenge of measuring cognitive states “in the moment” extends beyond memory research and is central to other fields such as human factors, where researchers often aim to assess constructs like situational awareness or cognitive workload without disrupting task performance. For example, situation awareness probes have been shown to risk interfering with task flow (e.g., McGowan & Banbury, 2004), raising similar concerns to those addressed in the present study. Additionally, less intrusive methods of tracking attentional state, such as EEG, pupil dilation, or activation patterns in regions like the dorsolateral prefrontal cortex, could be useful alternatives to thought probes. Future work could integrate these physiological or neuroimaging-based approaches to triangulate moment-to-moment mind-wandering with minimal disruption to encoding processes.

In sum, the present study investigated whether including thought probes or other interruptions in a memory task causes reactivity that may bias results. Most prior work examining potentially reactive mind-wandering probes has focused on processing speed or other cognitive abilities besides memory (e.g., Arnicane et al., 2021; Wiemers & Redick, 2019). Here, we demonstrated that mind-wandering probes do not significantly impact overall memory performance (though there may be some impact on items immediately preceding or following a probe) or the temporal dynamics of episodic memory. Thus, the present study suggests that using mind-wandering probes introduces minimal unexpected bias into research designs such that these interruptions do not adversely affect or benefit memory performance, consistent with prior research focused primarily on other cognitive domains. For researchers concerned about the reactivity of mind-wandering probes, our findings suggest they can be used during encoding tasks without substantially biasing recall performance.

## Notes

1. Participants provided a response on 69% of math trials. The reported accuracy rate of 39% reflects all trials, including non-responses. When excluding trials where no response was given, accuracy rose to 57%, indicating moderate accuracy among those who submitted an answer within 7 s.
